# Secure approximation of edit distance on genomic data

**DOI:** 10.1186/s12920-017-0279-9

**Published:** 2017-07-26

**Authors:** Md Momin Al Aziz, Dima Alhadidi, Noman Mohammed

**Affiliations:** 1 Department of Computer Science, University of Manitoba, Winnipeg, Canada; 2 Zayed University, Dubai, United Arab Emirates

**Keywords:** Privacy of genomic data, Secure edit distance, Secure genomic sequence similarity, Genomic sequence similarity, Edit distance approximation on genomic data

## Abstract

**Background:**

Edit distance is a well established metric to quantify how dissimilar two strings are by counting the minimum number of operations required to transform one string into the other. It is utilized in the domain of human genomic sequence similarity as it captures the requirements and leads to a better diagnosis of diseases. However, in addition to the computational complexity due to the large genomic sequence length, the privacy of these sequences are highly important. As these genomic sequences are unique and can identify an individual, these cannot be shared in a plaintext.

**Methods:**

In this paper, we propose two different approximation methods to securely compute the edit distance among genomic sequences. We use shingling, private set intersection methods, the banded alignment algorithm, and garbled circuits to implement these methods. We experimentally evaluate these methods and discuss both advantages and limitations.

**Results:**

Experimental results show that our first approximation method is fast and achieves similar accuracy compared to existing techniques. However, for longer genomic sequences, both the existing techniques and our proposed first method are unable to achieve a good accuracy. On the other hand, our second approximation method is able to achieve higher accuracy on such datasets. However, the second method is relatively slower than the first proposed method.

**Conclusion:**

The proposed algorithms are generally accurate, time-efficient and can be applied individually and jointly as they have complimentary properties (runtime vs. accuracy) on different types of datasets.

**Electronic supplementary material:**

The online version of this article (doi:10.1186/s12920-017-0279-9) contains supplementary material, which is available to authorized users.

## Background

Similar Patients Query (SPQ) [1] is used to identify similar patients from a large number of medical sources. The similarity is measured based on the sequenced genomes of patients. Nowadays sequencing and interpreting genomic information is cheaper and easier than ever. However, executing SPQs has been seen as a double-edge sword. The results of executing SPQs will lead to a better diagnosis of diseases and early detection of certain diseases. On the other hand, executing SPQs raises some security and privacy concerns. DNA sequences include health and other information about patients and their families. The disclosure of such genomic sequences could harm patients from different perspectives such as affecting the employment and the education opportunities. What makes things more serious, are some federal laws to address privacy issues such as the Health Insurance Portability and Accountability Act (HIPAA) [2]. HIPAA is the United States’ legislation that provides data privacy and security provisions for safeguarding medical information. Accordingly, there is a desideratum to privately execute SPQs over genomic data.

Edit distance or Leveshtein Distance [3], which has been a popular metric of string similarity, can be defined as the minimum number of operations (insertions, deletions and substitutions) required to convert one string to another. This metric is widely used in different problems for its superior utility and accuracy over other string distance metrics such as hamming distance and Jaro-Winkler distance [4]. For human genomic data, edit distance seems to capture the requirement as we can find similar patients [1] based on genomic information. However, this superiority comes with a cost as edit distance is a quadratic time algorithm. That is, given two strings with *n* lengths, it requires *O*(*n* ^2^) operations to compute the edit distance; this is not acceptable for long string sequences. For this reason, edit distance problem has been studied over the years by the theoretical computer science community in order to find a better alternative, a faster algorithm [5, 6], or an approximation algorithm. Particularly, in human genomic data where we have billions of base pairs and genomic sequences are constructed with nucleotides (*A, T, G, C*), this algorithm falls short as most datasets contain millions of records. For this reason, other algorithms of string similarity to deal with genomic data have been proposed [7, 8]. These algorithms have been mainly diverged into two directions, either designing faster algorithms by bounding the algorithm or resorting to an approximation which is the approach that we adopt in this paper.

Privacy and time efficiency should be considered while computing the edit distance over human genomic data to find similar patients. Data owners are not wiling to share their genomic data in plaintext to researchers to avoid re-identification of patients [9, 10] and legal consequences [2]. Proper authentication and access control over these high volume of sensitive genomic data are ensured with time costly verification methods which often results in delays by several months [11].

In this paper, we propose a framework which captures these requirements by preserving the privacy of the query issued by a researcher and the genomic data owned by a data owner in a time efficient manner. In other words, our framework allows efficient approximation algorithm of string similarity over genomic data where the data owner cannot see the researcher’s query and the researcher cannot access the genomic data of the data owner. The proposed framework consists of two algorithms of approximating the edit distance over genomic data. The first one resorts to the concept of shingles [12] supported by private set intersection techniques [13]. The second one depends on the banded alignment [14, 15] implemented using garbled circuits [16, 17]. The contributions of this article can be summarized as follows:

- We propose an approximation of the edit distance based on shingles [12] and the Permutation-based Hashing Set Intersection (Phasing) [13]. A *k*-shingle for a genomic sequence can be defined as any substring of length *k* that can be found within the sequence. Shingles are generated for the sequences of the data owner and the sequence of the researcher. Phasing is then used to privately intersect the shingles of the researcher and the shingles of the data owner such that the query and the genomic data are obscured from the data owner and the researcher, respectively.
- We propose another algorithm of approximating the edit distance the preserves the privacy of the query and the genomic data using the banded alignment and garbled circuits. The banded alignment approximates the edit distance by reducing the number of the needed comparisons. To privately execute the banded edit distance, we resort to garbled circuits.
- We experimentally show that the first approximation algorithm is time-efficient whereas the second one is more accurate using different datasets. We also show that the first approximation can be applied before the second one because they have complimentary properties. Moreover, we compare these approximations with stat-of-the-art techniques [1]. Experimental results show that our proposed algorithms outperform existing techniques both in terms of efficiency and accuracy.

## Problem definition

Similar Patients Query [1, 18] mainly uses edit distance as a metric to measure the similarity between different genomic sequences. It allows researchers or health care professionals to retrieve similar genomic sequences based on a query sequence. For example, a new patient gets admitted and the physician is seeking for previous patients with similar genomic sequences. The history of previous patients will help the physician to come up with a definitive diagnosis in a timely manner.

**Fig. 1:**
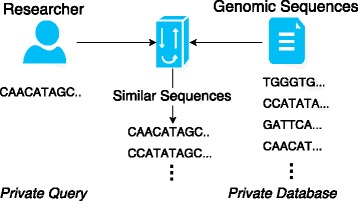
Problem architecture

The architecture of the proposed framework is shown in Fig. 1. It consists of two entities: a researcher and a data owner (i.e., hospital or genomic data storage). The researcher is working on a new disease but cannot reveal the subject’s sequence to the data owner and the data owner does want to disclose its genomic data to the researcher. Both parties need a protocol to interact with each other to determine similar sequences without disclosing the genomic sequences of their patients. The number of sequences revealed to the researcher through the private mechanism is predefined.

More formally, given a dataset of genomic sequences *G* *S*=*s*_1_,*s*_2_,…*s*_*n*_ owned by a data owner and a genomic sequence *s*_*q*_ provided by the researcher as a query predicate, the problem of similar patients query (SPQ) is to retrieve the top-*k* similar patients from *GS*, where the *k* sequences are determined according to the query sequence *s*_*q*_ and a similarity metric (i.e., edit distance). The retrieval should be conducted in a way such that the data owner cannot see *s*_*q*_ and the researcher cannot access any sequence in *S* other than the final output (i.e., top-*k* sequences).

## Preliminaries

In this section, we present an overview of the building blocks that are utilized in the proposed solution.

### Edit distance

A word over the finite alphabet *Σ* is a sequence *a*_*i*_,…,*a*_*n*_ of symbols where *a*_*i*_∈*Σ* for *i*=1,…,*n*. The empty word is denoted by *ε*. An edit operation is a pair (*a*,*b*) with *a*,*b*∈*Σ*∪{*ε*} and *a* *b*≠*ε*. The edit operation (*a*,*b*) is called an insertion if *a*=*ε*, a deletion if *b*=*ε*, and a substitution if *a*≠*ε*≠*b*. An edit operation is a basic step in transforming a word into another word. The meaning of the operations (*ε*,*b*), (*a*,*ε*), and (*a*,*b*) is to insert *b*, to delete *a*, and to substitute *a* by *b*, respectively. A cost *c*(*a*→*b*) is assigned to each edit operation (*a*,*b*). It is generally assumed that *c*(*a*→*b*)=1 and *c*(*a*→*b*)=0 for *a*≠*b* and *a*=*b*, respectively. An edit sequence *S* is a sequence of edit operations, *S*=((*a*_1_,*b*_1_),…,(*a*_*n*_,*b*_*n*_)),*n*≥1. The cost of an edit sequence *S* is defined as $C(S)= \sum _{i=1}^{n} c(a_{i},b_{i})$

#### **Definition 1**

The edit distance *d*(*X*,*Y*) between two words *X*,*Y* is defined as the minimum cost taken over all edit sequences that transform *X* into *Y*. That is *d*(*X*,*Y*)=*m* *i* *n*{*C*(*s*)|*s* is a sequence of edit operations transforming *X* into *Y*}.

For example, Let us assume that *X* and *Y* are genomic sequences such that *X*=*A* *T* *G* *C* and *Y*=*A* *T* *G* *G*. It takes one operation to convert *X* to *Y*. In other words, the edit distance is one. Wagner Fischer’s algorithm to compute the edit distance is shown in Algorithm 1 [19].



### Garbled circuits

A Garbled Circuit (GC) is a constant round protocol which allows any function to be securely computed between multiple parties. This concept was defined in 1982 by Yao [16] to solve “The Millionaire Problem”. After much optimization through the years [20], many implementations are currently available like ObliVM [25] or FastGC [17]. The millionaire problem explains the importance of garbled circuits in secure multiparty computations. Suppose two millionaires want to determine who is richer but they do not want to reveal their exact wealth. They initiate a GC between them and the result will be a boolean which denotes any single party’s value is greater than the other. One party (generator) generates the total circuit and keys whereas the other one (evaluator) evaluates it.

**Fig. 2:**
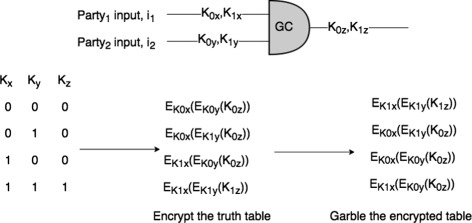
Garbled Circuits

Figure 2 shows an example of garbled circuit for AND gate. The generator picks two random keys for each wire such that one key corresponds to 0 and the other corresponds to 1 resulting 6 keys in total for each gate. After that the generator encrypts each row of the truth table by encrypting the output-wire key with the corresponding pair of input-wire keys. Then it randomly garbles the table, and sends it to the other party (evaluator) along with the key corresponding to its input bit ($E_{k_{0x}}$ if the input is 0 or $E_{k_{1x}}$ if the input is 1). The evaluator evaluates the circuit by performing an oblivious transfer [21] to get the key that corresponds to its input bit and then decrypts exactly one of the output-wire keys. The evaluator sends the generator the key for the final output wire and the generator informs the evaluator if it corresponds to 0 or 1. Oblivious transfer is another cryptographic protocol where the generator puts $E_{k_{0y}}$ or $E_{k_{1y}}$ as inputs and the evaluator picks its input from it. This protocol ensures that the evaluator does not learn the other input and the generator is unaware of evaluator’s pick. The beauty of the GC protocol is that only one row of the encrypted table will be decrypted by the evaluator to a proper value (with two keys).

### Threat model

We adopt the semi-honest model where both parties follow the protocol but may try to deduce additional information from the received messages. A protocol is private in a semi-honest environment if the view of each party during the execution of the protocol can be effectively simulated by a probabilistic polynomial-time algorithm knowing only the input and the output of that party [22]. Many protocols involve the composition of privacy-preserving subprotocols in which all intermediate outputs from one subprotocol are inputs to the next subprotocol. These intermediate outputs are either simulated given the final output and the local input for each party or computed as random shares. Using the composition theorem [22], it can be shown that if each subprotocol is privacy-preserving, then the resulting composition is also privacy-preserving. The semi-honest model is a realistic adversary model in the context of this paper where a level of trust among the parties can be ensured through a mutual legal agreement.



## Methods: Edit distance approximations over genomic data

In this section we discuss two different techniques to approximate the edit distance over genomic data. These approximation algorithms are detailed in the following subsections.

### Shingles with private set intersection

The first approximation algorithm consists of two steps. The first step depends on the concept of shingles [12, 23] whereas the second one depends on the Private Set Intersection (PSI) [13]. These two steps are summarized in Algorithm 2.

**Shingles.** Shingling [12, 24] is a technique used to identify lexically similar documents in data mining. For any string *S*, a *w*-shingle is a set where each item is a substring of length *w*. These items can be unique or might appear more than once (bag technique). In this paper, we only consider the unique property of the shingles.

#### **Example 1**

Consider one genomic sequence ‘CAACATAGCAAC’ and *w*=4, then the set of *4*-shingles will be {*CAAC*, *AACA*, *ACAT*, *CATA*, *ATAG*, *TAGC*, *AGCA*, *GCAA*}.

Notice that ‘*CAAC*’ appears twice in the sequence but only considered once when constructing the shingles. To the best of our knowledge, this is the first time this concept is used in privacy preserving computation of genomic data. It is particularly helpful for genomic sequences as we have only four nucleotides (*A*,*T*,*G*,*C*) to consider. In this step, the data owner and the researcher generate the *w*-shingles for the genomic sequences in the dataset and the genomic sequence in the query, respectively.

**Private Set Intersection (PSI)** It is a useful technique and is used in many real applications [13]. It addresses the problem of two parties who do not want to share their data but want to discover the common items between them. Formally,

#### **Definition 2**

Consider two different parties having two different sets *A* and *B* respectively. The output of a private set intersection only reveals the set *A*∩*B*={*x*:*x*∈*A*∧*x*∈*B*} while *A* and *B* are kept private from each party.

In this step, we adopt state of the art Permutation-based Hashing Set Intersection (Phasing) [13] to privately intersect the shingles of the researcher and the shingles of the data owner generated in the first step. The data owner does not share its data or see the query sequence from the researcher. The data owner gets the result of the intersected shingles and orders the records according to the number of matches with the intersection result. For example, if record 1 has 10 shingles in the intersection set whereas record 2 has 9 singles, then record 1 is more similar to the query sequence than record 2. The data owner picks out the top-*k* and sends them to the researcher. The process is stated in Algorithm 2.



### Banded alignment using garbled circuits

The second approximation algorithm depends on two concepts: the banded alignment [14] to compute the edit distance and garbled circuits [16, 17] to compute the banded edit distance in a privacy-preserving setting. The original Wagner Fischer’s algorithm detailed in Algorithm 1 has an average case running time of *O*(*n* *m*) where *n* is the number of sequences and *m* is the length of a genomic sequence. Since genomic sequences are generally long, running time *O*(*n* *m*) is not scalable for human genomes [1]. We adopt in this step a banded alignment [14] to reduce the runtime from *O*(*n* *m*) to *O*(*n* *b*) where *b* is a constant (band length). As outlined in Algorithm 3, we only compare each nucleotide from sequence *A* with a certain region *b* in the second sequence. Algorithm 1 has to calculate through both of the whole sequences to find its score.

To execute the banded edit distance detailed in Algorithm 3 in a private setting, we resort to garbled circuits [16, 25]. Due to privacy constraints, it is unwise to compare nucleotides at different positions using garbled circuits. The researcher can exhaustively find out the corresponding value in any given respectable position. This is why the banded edit distance is implemented using a garbled circuit where the final output is the edit distance between the sequences (see Section Security discussions for more discussion).

Garbled circuits are expensive time-wise especially if the data owner owns a large number of records. To overcome this deficiency, we apply the banded edit distance using garbled circuits after shingling and PSI as both approximation algorithms have complimentary properties.

### Joined approach

**Fig. 3:**
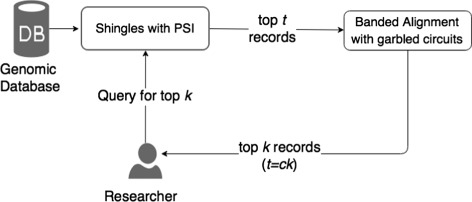
Execution order (second approximation)

In the joined approach, we use the first approximation (shingling and PSI) to reduce the search space for the second approximation as the first one is computationally much faster than the second one. Here the first method decreases the number of records that are used as an input to the second approximation by retrieving the top-*t* for a top-*k* query (*t*>*k*) as detailed in Fig. 3. The relation between *t* (the top-*t* records resulted from the first approximation) and *k* (the top-*k* that should result from the second approximation) is *t*=*c* *k* where *c* is a constant. The value of the constant *c* depends on the value of *k*, sequence length and dataset size. For example, if the dataset of the data owner contains 2000 records and the researcher is interested in the top-10 similar sequences, the first approximation reduces the number of records to 50 (*c*=5 and *t*=50) and then we will use these 50 records as an input to the second approximation to end up with the top-10 similar records (*k*=10). If the value of *k* is changed to 20, *t* becomes 100 (*t*=5×20) records.

Notice that if the number of the records in the dataset of the data owner is not large or the sequence lengths are small, then there is no need for the first approximation algorithm to decrease the number of the records as banded alignment over garbled circuit will be efficient enough. We further show these relations on Section Results.

To get the top-*k* after that, Algorithm 3 depends on the garbled circuit to compare two sequences. After that, the data owner orders the records of the dataset according to the values of the edit distance and sends the top-*k* records to the researcher.

## Results

In this section, we analyze the performance of the proposed approximation algorithms for privacy-preserving genomic data similarity problem. To simulate the real-life scenario, we placed the data owner and the researcher in a virtual machine with 4GB RAM. The reason behind this choice is that we are interested in computing the required time to securely execute both approximations with fixed network latency (5-10ms). The source code is available publicly at GitHub [26] for interested readers. We consider the following aspects in order to assess the efficiency of the proposed approximation algorithms.

- *Space complexity for shingles:* storage space needed to store the shingle dataset.
- *Accuracy analysis:* performance of the approximation algorithms measured against the original edit distance algorithm.
- *Runtime analysis:* time needed for preprocessing and to answer the researcher’s query.
- *Benchmarking:* accuracy and time comparison with a state of the art technique [1].

We used both real-life and synthetic datasets for evaluating our model. The real-life dataset is taken from the recent iDASH competition 2016 [27] where there were approximately 3000-4000 different SNPs from 500 different individuals. For better analysis, we generated synthetic data by accumulating the allele frequency of CHB, CHS, JPT and MXL populations from *1000genomes* dataset (August 2010 Release) [28] and generated 2000 genomic sequences with around 9000 SNPs each. Corresponding details about the datasets are presented in Table 1. The query sequence length for Database 1 is (3465) and specified by the iDASH competition 2016 [27]. For Database 2, the query sequence length is (9000-10,000). Actually, the 50 query sequences were generated while generating Dataset 2. In other words, we generated 2050 sequences such that 50 were assigned for the query and the rest constructed the dataset We will call the real-life dataset taken from iDASH2016 and the synthetic dataset generated from *1000genomes* Dataset1 and Dataset2, respectively throughout the rest of the paper.

**Table 1:** Dataset consideration

Parameters	Dataset 1	Dataset 2
Number of records (*n*)	500	2000
Sequence length (*l*)	3400-3500	9000-10000
Number of queries	1	50
Query length	3461	9000-10000
Data size (MB)	1.65	17.2
Data source	iDash 2016 [27]	Generated

### Space complexity for shingles

As transforming a genomic dataset to a shingle dataset will be space exhaustive, we need to analyze the space requirements for different shingle sizes. We only consider unique strings when transforming the original genomic dataset to shingles dataset. For example, if there are *n* genomic sequences each with *l* length, then the size of the dataset is *n*×*l*. If we consider fixed size *w*-shingles (i.e., *w*=5) then we need to construct a *r*×*w* dataset where there are *r* unique shingles each with *w* length. For example, if *w*=2, we have only 4^2^ possible shingles (*A* *A*,*A* *T*,*A* *C*,*A* *G*,…) since sequences are constructed with 4 nucleotides (*A, T, G, C*). This converts a *n*×*l* genomic dataset to a 16×2 shingle dataset. However this transformation will be expensive for large values of *w*.

Theoretically, the number of unique shingles (4^*w*^) should grow exponentially as the size of *w* increases. Nevertheless, due to the high repetitions in genomic sequences, this quantity is much smaller for practical application scenarios. As shown in Table 2, if *w*=10 then we have 354,457 shingles from a dataset of 3,000 records (9,000-10,000 length) where theoretically we should have 4^10^=1,048,576. This results in a shingle dataset of 4.05 MB whereas the size of the original dataset with sequences was 17.718 MB. Larger values of *w* increases the size of the shingle dataset as shown in Table 2.

**Table 2:** Relationship between the shingle dataset size and the number of unique shingles for different shingle size (*w*)

Shingle size *w*	Unique shingles	Shingle dataset size (MB)
5	1024	0.007
10	354,457	4.05
15	1,383,525	22.4
20	2,927,918	61.4

### Accuracy analysis

As we are proposing two approximation algorithms, we analyze their accuracy separately and jointly. Here the accuracy is defined as,

$$\begin{array}{*{20}l} accuracy &= \frac{\text{\# of match in a top-k query} }{\text{\# of positives from edit distance}}\\ &=\frac{N_{TP}}{N_{P}}=\frac{N_{TP}}{N_{TP}+N_{FN}} \end{array} $$

where *T* *P*,*F* *N*,*P* are true positives, false negatives and positives, respectively. In general, this accuracy (also known as true positive rate, sensitivity or recall) denotes how many records are positives for both the approximation algorithms and the original edit distance algorithm. For example in a top-*3* query, we have records {1000,1010,505,1101} as an output from the edit distance algorithm where the records 505 and 1101 have the same distance and ranked 3rd. Similarly, from our approximation algorithm, we have the rank as {1000,1010,505,202} which will lead the accuracy to be $\frac {3}{4}=75\%$. Some further analysis and explanation are available in the Additional file 1 document as well.

The first approximation algorithm using shingles and PSI is much accurate when the dataset is small (i.e., Dataset 1). While for larger datasets, this method falls short and we need the banded alignment algorithm to obtain good accuracy. They can also be used in conjunction or jointly.

**Fig. 4:**
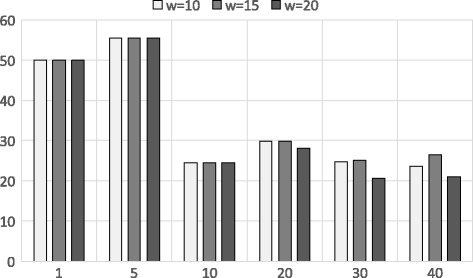
Accuracy of shingling and PSI approximation using Dataset 1. X-axis shows different *k* values (top-*k*) and Y-axis shows the accuracy for different *w* values

In Fig. 4, we depict the accuracy of the shingling and PSI approximation using Dataset 1. The dataset had 500 records and short sequences (around 3000 each). The performance of the proposed approximation (shingles and PSI) algorithm is measured against the original edit distance algorithm. The optimal value of *w* is (*l* *o* *g*_|*Σ*|_ *l*) [15] where *l* is the sequence length and |*Σ*|=4 for genomic sequences. However, as lower *w* values resulted in higher false positives, those are not showed here for brevity. However, more graphs are shown in the Additional file 1 regarding this issue.

**Fig. 5:**
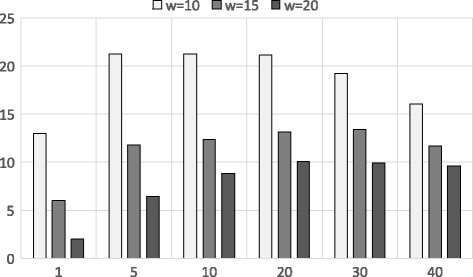
Accuracy of shingling and PSI approximation using Dataset 2. X-axis shows different *k* values (top-*k*) and Y-axis shows the accuracy for different *w* values

Aforementioned, this method has some shortcomings when dealing with Dataset 2 where we have longer sequences. In Fig. 5 we show this deficiency as the accuracy ranges in 2−13*%* for top-1 queries. This is due to the higher sequence lengths and numbers as shingle matches cannot efficiently represent the original edit distance.

**Fig. 6:**
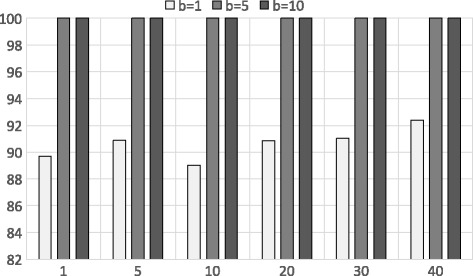
Accuracy of the banded alignment using Dataset 2. X-axis shows different *k* values (top-*k*) and Y-axis shows the accuracy for different band values *b* values

Due to this deficiency from shingles and PSI approximation algorithm, we switch the other technique to approximate edit distance which is more accurate for longer sequences. The accuracy of our banded alignment is showed in Fig. 6. The accuracy of this method is impeccable due to the resemblance with the original edit distance and lower dimension of data. However there is a certain cost involved in executing the banded alignment over a garbled circuit (to provide security) which results in longer run times. We further elaborate this notion in the upcoming run time analysis section.

**Fig. 7:**
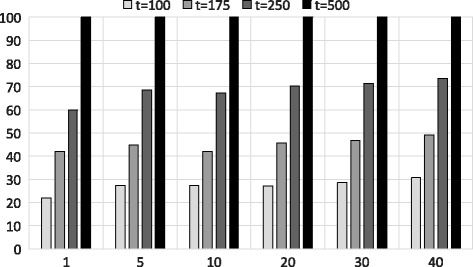
Accuracy of the banded alignment after shingles and PSI method using Dataset 2. X-axis shows different *k* values (top-*k*) and Y-axis shows the accuracy for different *t* values

Figure 7 shows the accuracy for both approximation algorithms joined according to Fig. 3. Here, we use the top-*t* outputs from the first approximation algorithm as an input to the banded alignment to end up with the target top-*k* results. It is clear from Fig. 7 that larger values of *t* returns better accuracy. On the other hand, larger values of *t* has a negative impact on the running time as demonstrated in the subsequent section. The accuracy in Fig. 6 is certainly better than the accuracy in Fig. 7. However, we proposed the combining of both approximations as shown in Fig. 3 because this reduces the execution time. We have further discussed these issues in the Additional file 1.

### Runtime analysis

We show in Table 3 a summary of the running time of both private approximation algorithms along with other insecure techniques. We also give the time required for the state of the art work conducted by Wang *et al.* [1] as it provides a solid benchmark for assessing runtime. The benchmarking is done using Dataset 2. The first approximation (shingling and PSI) is the fastest. The banded alignment takes longer since it depends on the sequence length of the query due to the runtime *O*(*n* *b*).

**Table 3:** Running time analysis (top-10 queries with *k*=10,*c*=5(*t*=*c* *k*),*w*=10, and *b*=5)

Dataset	Method	Preprocessing	Query
		Time (s)	Time (s)
Dataset 1	Plain Edit Distance	0	23
Dataset 1	Shingles with PSI	18	5
Dataset 1	Protocol 1 [1]	5.7	585
Dataset 1	Protocol 2 [1]	5.7	511
Dataset 2	Plain Edit distance	0	930
Dataset 2	Protocol 1 [1]	61	3049
Dataset 2	Protocol 2 [1]	61	2800
Dataset 2	Shingles with PSI	181	108
Dataset 2	Shingles with PSI +	181	730
	banded alignment		

**Fig. 8:**
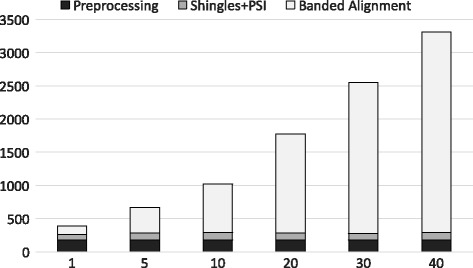
Run time analysis using Dataset 2. X-axis shows different *k* values (top-*k*) and Y-axis shows the run time (in seconds) for different approximations where *b*=5 and *c*=5

This concern can be further elaborated in Fig. 8 where we show the run time for both approximation algorithms with different band sizes and c. The noticeable aspect of Fig. 8 is that the running time of the joined approach has a linear relationship with the value of *k*. If *k* is increased, *t* (the input of the banded alignment (*t*=*c* *k*)) will be increased and accordingly the running time will be increased. This is the primary reason behind using the shingle approach before the banded alignment in the joined approach as it reduces the search space in a constant time for the banded alignment for a large dataset. Also, it is clear that the preprocessing time is a one time cost and depends on the genomic database size which we can neglect. However, the banded alignment over garbled circuit can be much faster under some security assumptions which we explain in 2. The run time for Dataset 1 is provided in the S Additional file 1.

### Benchmarking

**Fig. 9:**
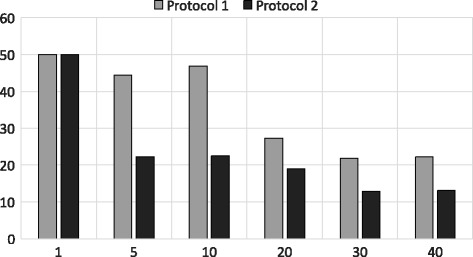
Accuracy of Protocol1 and Protocol2 [1] using Dataset 1. X-axis shows different *k* values (top-*k*) and Y-axis shows the accuracy for both protocols

**Fig. 10:**
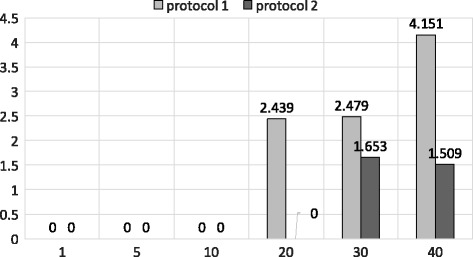
Accuracy of Protocol1 and Protocol2 [1] using Dataset 2

In Figs. 9 and 10 we show the performance of the state of the art approximation algorithm proposed by Wang et al. [1] using Dataset 1 and Dataset 2, respectively. Both protocols presented in the paper of Wang et al. have high accuracy using Dataset 1 for top- {1,5,10,20,30,40} queries which resemble our PSI and shingle based approach. It is noteworthy that we take much less time to achieve similar accuracy (24*s* vs 585*s*). However, this accuracy drops for longer sequences as shown in Fig. 10 for Dataset 2. This clearly shows the benefit of our second approximation algorithm using the banded alignment technique. Thus, our joined approach achieves a good balance between accuracy and runtime.

## Security discussions

In this section, we elaborate some of our design choices and discuss the limitations of the proposed methods.

### Security of private set intersection methods

In addition to Phasing algorithm [13], there are a number of other private set intersection techniques [29]. Among these, we experimentally evaluated the basic hashing based method [30], Diffie-Hellman based protocol [31] and permutation based hashing method [13]. We found that only the hashing based method has a better performance than the Phasing algorithm. We did not opt for the hashing method [13] because an active adversary can run a brute force algorithm on a specific shingle size (*w*). This will eventually reveal the query sequence (or genomic data) as the data owner (or the researcher) can reconstruct the sequence from shingles. Therefore, we use the Phasing algorithm [13] where such attack is not possible.

### Banded alignment in garbled circuit

In the banded alignment, we implemented the whole algorithm using a garbled circuit (GC). This design choice is due to the leakage consideration of individual position comparisons of the edit distance algorithm. In the original edit distance algorithm, characters are matched one at a time at different positions of the sequences of the query and the dataset. If a researcher is allowed to query the genomic dataset and individual comparisons are done using a GC, then s/he can exhaustively find out the corresponding value in any given respectable position as there are only 4 possible values (A, T, G, C). However, in our method, the whole iteration of the computation is done inside a garbled circuit and it outputs only the final result of the edit distance. Thus, our banded alignment protocol allows a researcher and a data owner to obliviously calculate the distance between two strings without leaking any further information.

### Joined approximation

The output of the joined approximation is the top-*k* sequences given a target query and these *k* sequences are public and the output of the protocol. In the joined approximation, the data owner knows the *t* records which are the output of the shingling and PSI. As these *t* records are not revealed to the researcher, it does not violate the security requirement. Also, it does not reveal any additional information to the data owner as *t* sequences are more general than the final *k* sequences.

## Related work

One of the primary works in the domain of privacy preserving genomic sequence similarity is conducted by Jha *et al.* [32]. In their paper, they showed three different protocols which can replicate the original edit distance algorithm over a garbled circuit. However, due to the performance of the garbled circuit available that time, it took around 40 seconds for computing the edit distance between two sequences where the length of each one of them is 25. After the proposal of the fully homomorphic encryption (FHE) by Gentry [33], edit distance was also proposed to be homomorphically computed via lattice encryption by Cheon *et al.* [34]. However, due to the current state of FHE, the scheme is still inefficient as it takes 16.4 seconds to compute a 8×8 block of dynamic programming. As the crypto behind the FHE advances and improves, we might see a better usage of this in the future.

The closest research to ours is conducted by Wang *et al.* [1]. The research addressed the problem of approximating the original edit distance in a realistic setting. The method used a public reference genomic sequence to compute an approximation of the edit distance between two strings. However, the selection of a public reference leaks some information about the underlying data distribution. Moreover, it affects the accuracy as the computation is done according to a reference. In a more recent work, Shimzu *et al.* [35] proposed the usage of Burrows-Wheeler transformation for finding target queries on a genomic dataset. The problem addressed in this paper [35] is different, although closely related, as it does not answer secure string similarity for genomic data. There are also some other related studies such as [36–38] which address approximating or securely computing edit distance. Some of these are summarized in Table 4.

**Table 4:** Chronological development of privacy preserving genomic data similarity methods

Authors	Year	Data (*n*×*m*)	Time (s)	Principal method
Jha et al. [32]	2008	25×25	<40	Smith-Waterman
Wang et al. [38]	2009	400×400	28.5	Custom
				protocols
Wang et al. [1]	2015	2000×9000	2800	Private set
				difference with
				a reference
				sequence
Cheon et al. [34]	2015	8×8	16.4	Homomorphic
				encryption
Shimzu et al. [35]	2016	2184 genomes	4-10	Burrows-Wheeler
				transform

## Conclusion

Securely computing edit distance between human genomes have become very important in medical and public health domains. We have proposed novel techniques to privately approximate the edit distance on human genomes. We have implemented these techniques and experimental results show that the proposed methods are accurate and time-efficient, and performs better than existing methods.

## Additional file

Additional file 1 Contains further analysis of the solution mechanisms. (PDF 243 kb)
