# Supplementary material for: Secure approximation of edit distance on genomic data
Source: BMC Med Genomics. 2017 Jul 26;10(Suppl 2):41. doi: 10.1186/s12920-017-0279-9 (PMC5547448; doi:10.1186/s12920-017-0279-9)
Supplement: Additional file 1 — Contains further analysis of the solution mechanisms. (PDF 243 kb) [file 12920_2017_279_MOESM1_ESM.pdf]

Secure Approximation of Edit Distance on Genomic Data: Supplementary Document

1 Tradeoff in Joined Approach

There is a tradeoff between accuracy and execution time while considering the joined approximation approach. Shingle-based approximation is computationally inexpensive while the banded alignment using Garbled Circuit is more accurate. This is why the parameter t is utilized to bound this tradeoff as higher values of t will result in more accurate but slower execution. However for smaller values of t after the first approximation, the search is only limited to the top-t using the banded alignment. The records, which are similar but do not show up in the top-t, are not considered and cause the false negative results.

2 Further Analysis

Some of the analysis on different parameters such as band size, shingle length, different values for c are provided here as well as their effect on different metric of accuracy.

2.1 Accuracy

For further analysis about the accuracy of the proposed approximations, we describe some notations used in the context of similarity search:

- True Positive (TP): records which are similar and also returned as similar by the approximation algorithm.
- False Positive (FP): records which are not similar but returned as similar by the approximation algorithm.
- True Negative (TN): records which are not similar and also are not returned as similar by the approximation algorithm.
- False Negative (FN): records which are similar but are not returned as similar by the approximation algorithm.

We will also need some metrics such as:

$$Recall = \frac{N_{TP}}{N_{TP} + N_{FN}}$$

$$Precision = \frac{N_{TP}}{N_{TP} + N_{FP}}$$

$$Fallout = \frac{N_{FP}}{N_{FP} + N_{TN}}$$

As we showed the recall values or true positive rates for propose algorithms along with the benchmarking, here we are more interested in the precision and the false positive rate (fallout rate).

2.1.1 Recall

As we provided some figures about the recall (accuracy) or True Positive Rates (TPR) in the main paper, here we show some more which may appear missing in the main paper. The true positive rates for the banded alignment and the joined approximation on Dataset 1 would be the point of interest here. In Figure 1 and Figure 2 we show the high true positive rates for the banded alignment and the joined approximation (banded alignment after PSI-Shingle approach) using Dataset 1, respectively. Figure 11 shows the true positive rates of shingling and PSI approximation using Dataset 1 for different low w values ($w = \{4, 5, 6\}$).

2.1.2 Precision

Precision denotes that a sequence which is returned as an output from the approximation algorithm is actually a similar one. In other words, higher precision will indicate better performance as this is one of the general requirement of any algorithm. We show the precision of the PSI-Shingle approach using Database 1 and Database 2 in Figure 3 and Figure 4, respectively. The PSI-Shingle approach has less precision using Dataset 2; however, it outperforms Wang *et al.*'s Protocol1. In Figure 5, we show the precision of the banded alignment using Dataset 2. From the figures, we can see that the PSI-Shingle and the banded alignment perform well using Dataset 1 and Dataset 2, respectively.

2.1.3 False Positive Rate or Fallout

In a ranked query, false positives are those records which do not belong to the top-k results but appear as a result. For example, suppose a genomic sequence is not ranked among the top-10 nearest sequences according to the edit distance metric. However, our approximation methods output that record in the top-10 allowing this to be treated as a false positive. This analysis is much useful for understanding the real accuracy of any approximation method as it reveals the

number of wrong answers that are included in the result of an approximation.

In Figure 6 we show the false positive rate ($\frac{N_{FP}}{N_{FP+TN}}$) of the first approximation using Dataset 1 where N_{FP} represents the number of false positives and N_{TP} represents the number of true positives. It shows that for a small value of w (i.e., $w = 5$), there are many unwanted false positives. For $w = \{10, 15, 20\}$ the false positives are rather less in quantity which is desirable. Figure 7 shows the perfect FPR which is expected for the banded alignment using Dataset 2. For $b = \{1, 5, 10\}$ the FPR is 0 for any top-k query. Figure 8 shows the FPR for the joined approximation. Figure 12 shows the fallout of shingling and PSI approximation using Dataset 1 for different low w values ($w = \{4, 5, 6\}$).

2.2 Run Time

We analyze the running time for Dataset 1 in Figure 9. Here the effect of using the banded alignment can be further explained. As we picked $c = 5$, a top-5 query ($k = 5$) will require the top-25 records ($t = c \times k = 5 \times 5$) from the PSI-Shingle approach. These (25) records will be used as an input to the banded alignment. This is why higher values of k increases the run time. For example, a top-40 query in this case results in 400 banded alignment executions over garbled circuit which take around 2436 seconds.

We also provide the time analysis for different values of the band size (b). large values of b will slow down the banded alignment over GC because large values of b will result in more computations. Figure 10 shows the query time for $b = 20$ where it takes 4199 seconds to do a top-40 query. Also it is noteworthy that for $b = 5$, it takes 2436 seconds. We do not provide the time analysis for a fixed value of t in joined approximation because the run time will be constant in this case.

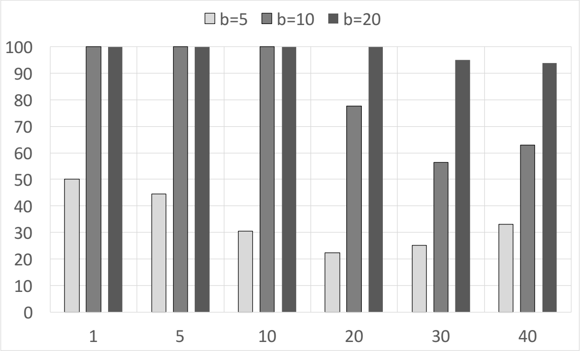

Figure 1 TPR (banded alignment) using Dataset 1. X-axis shows different k values (top-k) and Y-axis shows the rate for different b values

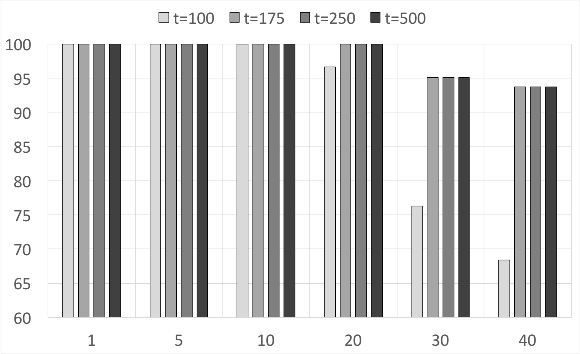

Figure 2 TPR (banded alignment after shingling and PSI method) using Dataset 1. X-axis shows different k values (top-k) and Y-axis shows the rate for different t values

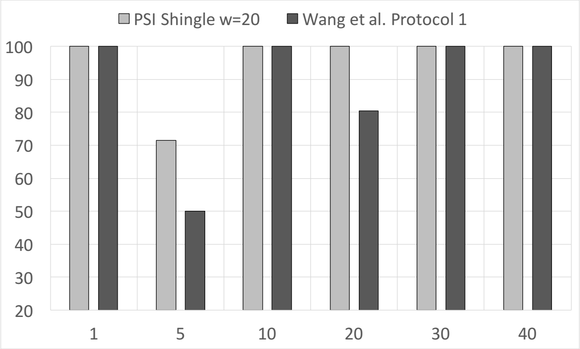

Figure 3 Precision of shingling and PSI approximation using Dataset 1. X-axis shows different k values (top-k) and Y-axis shows the rate for different approaches

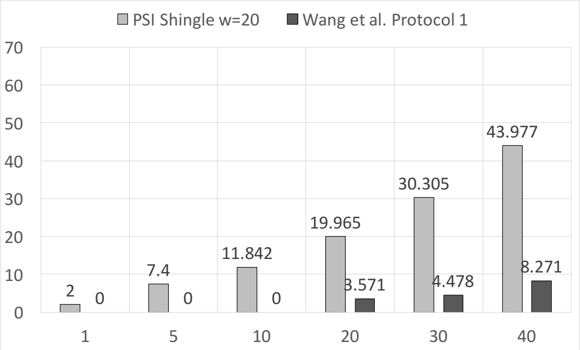

Figure 4 Precision of shingling and PSI approximation using Dataset 2. X-axis shows different k values (top-k) and Y-axis shows the rate for different approaches

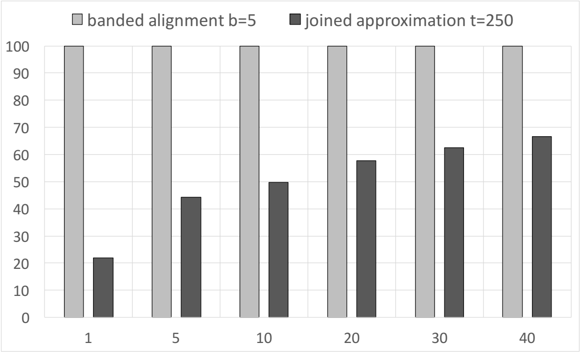

Figure 5 Precision of banded alignment using Dataset 2. X-axis shows different k values (top-k) and Y-axis shows the rate for different approaches

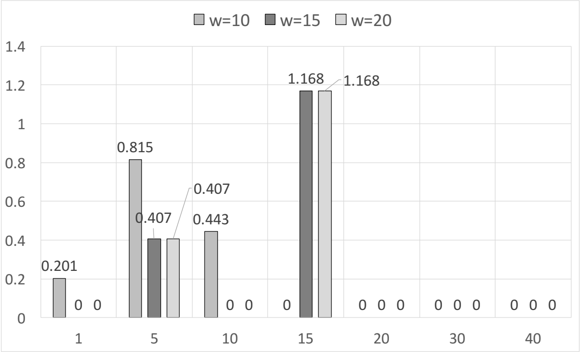

Figure 6 False positive rate of shingling and PSI approximation using Dataset 1. X-axis shows different k values (top-k) and Y-axis shows the ratio for different w values

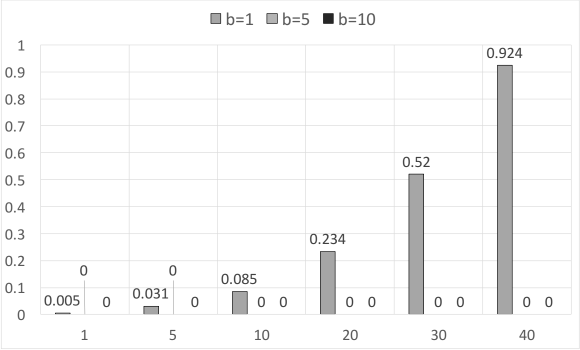

Figure 7 False positive rate of the banded alignment using Dataset 2. X-axis shows different k values (top-k) and Y-axis shows the ratio for different t values

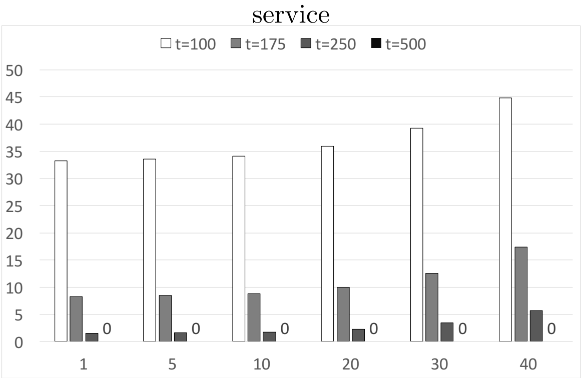

Figure 8 False positive rate of the banded alignment after shingles and PSI method using Dataset 2. X-axis shows different k values (top-k) and Y-axis shows the ratio for different t values

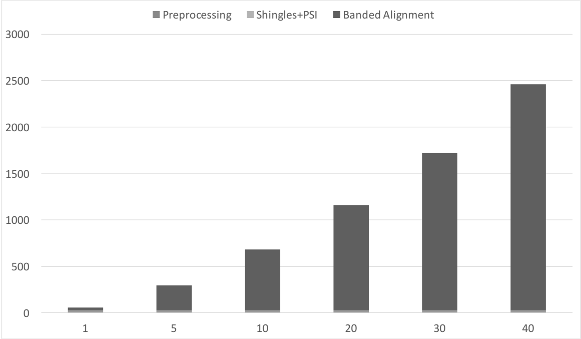

Figure 9 Run time analysis using Dataset 1 X-axis shows different k values (top-k) and Y-axis shows the run time (in seconds) for different approximations where $b = 5, c = 10, t = ck$

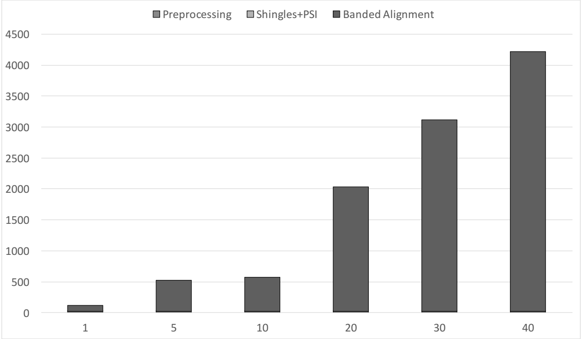

Figure 10 Run time analysis using Dataset 1. X-axis shows different k values (top-k) and Y-axis shows the run time (in seconds) for different approximations where $b = 20, c = 5, t = ck$

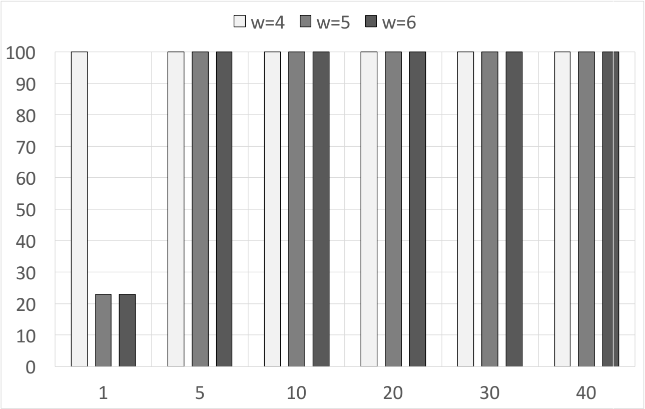

Figure 11 True positive rate of shingling and PSI approximation using Dataset 1. X-axis shows different k values (top-k) and Y-axis shows the ratio for $w = \{4, 5, 6\}$ values

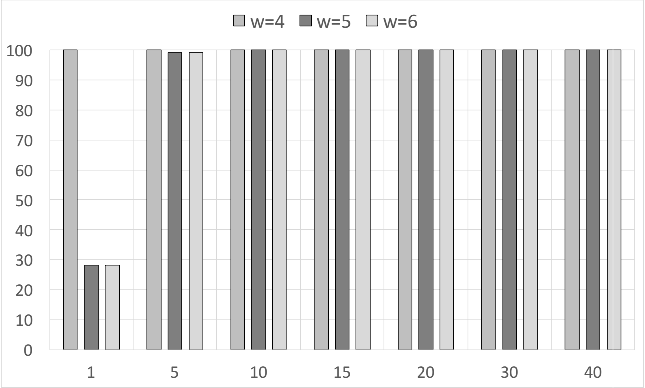

Figure 12 False positive rate of shingling and PSI approximation using Dataset 1. X-axis shows different k values (top-k) and Y-axis shows the ratio for $w = \{4, 5, 6\}$ values
